# Health, lifestyle and occupational risks in Information Technology workers

**DOI:** 10.1093/occmed/kqaa222

**Published:** 2021-01-30

**Authors:** D Lalloo, J Lewsey, S V Katikireddi, E B Macdonald, E Demou

**Affiliations:** 1 Healthy Working Lives Group, Institute of Health and Wellbeing, University of Glasgow, Glasgow, UK; 2 Health Economics and Health Technology Assessment, Institute of Health and Wellbeing, University of Glasgow, Glasgow, UK; 3 MRC/CSO Social and Public Health Sciences Unit, Institute of Health and Wellbeing, University of Glasgow, Glasgow, UK

**Keywords:** Behaviours, computer professionals, information technology, lifestyle, occupational health, UK Biobank

## Abstract

**Background:**

Information technology (IT) and the IT workforce are rapidly expanding with potential occupational health implications. But to date, IT worker health is under-studied and large-scale studies are lacking.

**Aims:**

To investigate health, lifestyle and occupational risk factors of IT workers.

**Methods:**

We evaluated self-reported health, lifestyle and occupational risk factors for IT workers in the UK Biobank database. Using logistic regression, we investigated differences between IT workers and all other employed participants. Regression models were repeated for IT worker subgroups (managers, professionals, technicians) and their respective counterparts within the same Standard Occupational Classification (SOC) major group (functional managers, science and technology professionals, science and technology associate professionals).

**Results:**

Overall, 10 931 (4%) employed participants were IT workers. Compared to all other employed participants, IT workers reported similar overall health, but lower lifestyle risk factors for smoking and obesity. Sedentary work was a substantially higher occupational exposure risk for IT workers compared to all other employed participants (odds ratio [OR] = 5.14, 95% confidence interval [CI]: 4.91–5.39) and their specific SOC group counterparts (managers: OR = 1.83, 95% CI: 1.68–1.99, professionals: OR = 7.18, 95% CI: 6.58–7.82, technicians: OR = 4.48, 95% CI: 3.87–5.17). IT workers were also more likely to engage in computer screen-time outside work than all other employed participants (OR = 1.42, 95% CI: 1.35–1.51).

**Conclusions:**

Improved understanding of health, lifestyle and occupational risk factors from this, the largest to date study of IT worker health, can help inform workplace interventions to mitigate risk, improve health and increase the work participation of this increasingly important and rapidly growing occupational group.

**Key learning points**

**What is already known about this subject:**

- The advancement of information technology has seen a rapid growth in the information technology workforce, potentially with substantial occupational health implications, yet there is a paucity of formal research on information technology worker health and well-being, particularly within Europe and North America.
- Most studies to date are small-scale, mainly within a single sector/company, making it hard to assess representability across this varied workforce and have largely neglected risks related to non-communicable diseases, despite their substantial disease burden.

**What this study adds:**

- Information technology workers have substantially higher exposures to sedentary work than both all other employed UK Biobank participants and comparator groups with similar occupational classifications.
- Compared to all other employed UK Biobank participants, information technology workers had lower levels of lifestyle risk factors for smoking and obesity; but higher computer screen-time outside work.
- This study fills a knowledge gap of risks related to non-communicable diseases in information technology workers.

**What impact this may have on practice or policy:**

- Improved understanding of health, lifestyle and occupational risk factors of this large and varied information technology worker population can help inform workplace interventions, to mitigate risk, improve health and increase work ability in information technology workers.
- This study sets a benchmark for large-scale information technology worker health studies.

## Introduction

Advancement of information technology (IT) has seen a rapid growth in the IT workforce [1], potentially with substantial occupational health (OH) implications. Given their pivotal role in economic and business development globally [2,3] and their increasing size, reliable data and research on this occupational group are essential, to help plan services, meet IT worker needs and establish areas of unmet OH need. To date, robust data and health and well-being research on this occupational group are lacking [2,3].

Defining an IT worker is complex and challenging [2,3]. The Information Technology Association of America (ITAA) uses a broad definition: any skilled worker who performs any function related to IT [3]. In essence, IT workers develop and maintain computer systems, and should be distinguished from other professional groups who use computers as part of their jobs. IT worker roles vary and include: software, hardware and network design and management, data management (storage, administration, retrieval, processing and protection) and helpdesk assistance [2,3]. Big data and artificial intelligence have become important functions as has information security, particularly with the shift to ‘cloud’ data storage.

IT jobs are not exclusively located within the IT industry, i.e. the industry that develops and sells IT software, services, systems and devices [3]. Given the rapid integration of IT into business, education, healthcare, industry and homes, IT workers are distributed across multiple sectors (public, private, government, education) and because of this dispersion, identifying and studying this IT worker group has proven difficult [3].

There is a paucity of formal research on IT worker health and well-being. Given the IT industry shift to Asia (due to cost savings and increased workforce availability) [4], IT worker health studies predominantly stem from these countries, where lifestyle, culture and working conditions are different and therefore not generalizable. There are only few European [5–8] and US [9,10] studies, two of these [5,9] briefly addressing health and lifestyle factors and the remainder, evaluating stress/burnout. Most IT worker studies to date are small-scale and limited to employees working in IT companies. They are usually set within a single company or sector, making it hard to assess representability across this varied workforce and they typically focus on three work-related outcomes: musculoskeletal problems, visual strain/fatigue and stress/psychological issues [11–14].

Research has largely neglected risks related to non-communicable diseases (NCDs) among IT workers, despite their substantial disease burden. While it has been surmised that high work stress, sedentary work and long working hours result in unhealthy behaviours and lifestyle choices [15,16], few IT worker studies have considered health behaviours and lifestyle risk factors [5,9,11,16–18] or evaluated these as outcomes. Many focus on sleep [5,11,18] and are limited to one or two risk factors, with a fuller, broader picture and robust assessment lacking. To our knowledge, there are no large-scale studies, and specifically, no UK-based studies assessing health behaviours and lifestyle factors of this growing workforce.

Improved understanding of occupational risk factors and exposures, even at sector level, can help inform workplace interventions to mitigate risk and reduce NCDs, thereby improving health and work participation. Our aim therefore was to evaluate health, lifestyle and occupational risk factors of IT workers compared with the general working population, other similar comparable occupational groups and within IT worker subgroups, in the UK Biobank.

## Methods

We analysed cross-sectional baseline data from the UK Biobank cohort study. Between 2006 and 2010 just over 500 000 participants from the general population aged 40–69 years were recruited into this study. This entailed touch-screen questionnaire completion and face-to-face interviews with physical and biological sample measurements described in detail elsewhere (https://www.ukbiobank.ac.uk/).

Baseline assessment included socio-demographic information (age, sex, ethnicity, socio-economic status using the Townsend score, household annual income, professional qualifications, current employment), self-reported health behaviour and lifestyle data (smoking status, alcohol consumption, physical activity, sleep, self-rated health perception, TV-viewing time, computer screen-time outside work) and physical health measurements (including height, weight, body mass index [BMI]). Employment status was recorded for 99% of participants alongside basic occupational exposure data including: whether the job involves mainly walking or standing, working week duration and work/job satisfaction.

Those ‘currently employed’ were interviewed by trained Biobank operators to gather job description information including, job title, job tasks and industry type. Following a tree structure algorithm, the operators coded the jobs using the four-digit Standard Occupational Classification (SOC) V.2000. A number (*n* = 18 322) of job descriptions could not be coded via the algorithm. These were subsequently SOC coded using the Computer Assisted Structure COding Tool (CASCOT) described in detail elsewhere [19] and were included in our analyses. Participants not employed were excluded.

All outcome variables were dichotomized into low or high risk based on current clinical and health guidelines, where available (see footnotes 1–4 in Table 2). Physical activity was based on the International Physical Activity Questionnaire (IPAQ) short form, converted into metabolic equivalents (MET min/week) and categorized into adequate (≥600 MET min/week) or inadequate (<600 MET min/week) [20]. Implausible values, i.e. total daily physical activity exceeding 24 h, were recorded as missing in our analyses. In the absence of available guidelines for adults, applying evidence on screen-time limits for adolescents and teens [21], total screen-time and its constituent categories (i.e. computer screen-time outside work and TV-viewing time), were dichotomized into ≤2 or >2 h/day.

**Table 2.** Lifestyle and work characteristics (A) in IT workers compared to ‘all other employed’ participants in the UK Biobank and (B) within IT worker subgroups

	A		B		
	All other employed, *n* (%)	All IT workers, *n* (%)	IT managers, *n* (%)	IT professionals, *n* (%)	IT technicians, *n* (%)
Total *n* (%) 287 151 (100)	276 220 (96)	10 931 (4)	3698 (1)	5756 (2)	1477 (0.5)
BMI (kg/m^2^)^1^					
<25	94 930 (34)	3684 (34)	1144 (31)	2047 (36)	493 (33)
≥25	180 207 (65)	7219 (66)	2548 (69)	3693 (64)	978 (66)
Missing**	1083 (0)	28 (0)	6 (0)	16 (0)	6 (0)
Smoking status					
Never smoker	157 515 (57)	6832 (63)	2265 (61)	3682 (64)	885 (60)
Previous/current smoker	117 923 (43)	4082 (37)	1428 (39)	2065 (36)	589 (40)
Missing**	782 (0)	17 (0)	5 (0)	9 (0)	3 (0)
Alcohol consumption^+^ (units/week)^2^					
≤14	56 287 (20)	2198 (20)	714 (19)	1162 (20)	322 (22)
>14	138 523 (50)	6247 (57)	2264 (61)	3274 (57)	709 (48)
Missing**	81 410 (30)	2486 (23)	720 (20)	1320 (23)	446 (30)
Physical activity (MET min/week)					
<600	26 134 (10)	1429 (13)	502 (14)	748 (13)	179 (12)
≥600	117 929 (43)	4662 (43)	1597 (43)	2454 (43)	611 (41)
Missing**	132 157 (48)	4840 (44)	1599 (43)	2554 (44)	687 (47)
Total screen-time^$^ (h/day)					
≤2	137 765 (50)	6054 (55)	2113 (57)	3240 (56)	701 (48)
>2	133 446 (48)	4748 (43)	1553 (42)	2440 (42)	755 (51)
Missing**	5009 (2)	129 (1)	32 (1)	76 (1)	21 (1)
Computer screen-time outside work (h/day)					
≤2	245 464 (89)	9115 (83)	3146 (85)	4727 (82)	1242 (84)
>2	26 821 (10)	1699 (16)	522 (14)	958 (17)	219 (15)
Missing**	3935 (1)	117 (1)	30 (1)	71 (1)	16 (1)
TV viewing (h/day)					
≤2	154 493 (56)	7174 (66)	2454 (66)	3891 (68)	829 (56)
>2	120 298 (44)	3740 (34)	1240 (34)	1859 (32)	641 (43)
Missing**	1429 (1)	17 (0)	4 (0)	6 (0)	7 (1)
Sleep (h/day)^3^					
≥7	204 098 (74)	8193 (75)	2724 (74)	4390 (76)	1079 (73)
<7	71 150 (26)	2729 (25)	973 (26)	1360 (24)	396 (27)
Missing**	972 (0)	9 (0)	1 (0)	6 (0)	2 (0)
Work/job satisfaction					
Happy	81 524 (30)	2829 (26)	953 (26)	1494 (26)	382 (26)
Unhappy	10 035 (4)	635 (6)	216 (6)	338 (6)	81 (6)
Missing**	184 661 (67)	7467 (68)	2529 (68)	3924 (68)	1014 (69)
Job involves walking/standing					
Always/usually/sometimes	182 962 (66)	2833 (26)	1080 (29)	1166 (20)	587 (40)
Never/rarely	92 868 (34)	8095 (74)	2617 (71)	4588 (80)	890 (60)
Missing**	390 (0)	3 (0)	1 (0)	2 (0)	0 (0)
Working hours per week^4^					
≤38	150 426 (55)	4741 (43)	1073 (29)	2795 (49)	873 (59)
>38	121 603 (44)	6120 (56)	2610 (71)	2912 (51)	598 (41)
Missing**	4191 (2)	70 (1)	15 (0)	49 (1)	6 (0)

**Includes ‘missing’, ‘do not know’ and ‘prefer not to answer’ responses.

^$^Total screen-time estimated as the sum of computer screen-time outside work and TV viewing (h/day).

^+^Recommended alcohol consumption guidelines changed in 2016 (i.e. following baseline data collection) from 21 units/week for women and 28 units/week for men to current thresholds of 14 units/week for men and women.

^1^BMI: underweight/normal weight (<25 kg/m^2^) or overweight/obese (≥25 kg/m^2^). National Institute for Health and Care Excellence. Obesity: Identification, Assessment and Management. 2014. https://www.nice.org.uk/guidance/cg189/chapter/1-recommendations#surgical-interventions.

^2^Alcohol consumption: ≤14 or >14 units/week. NHS Choices. Alcohol Units. 2015. http://www.nhs.uk/Livewell/alcohol/pages/alcohol-units.aspx.

^3^Daily sleep: adequate (≥7 h/day) or inadequate (<7 h/day). National Sleep Foundation’s sleep time duration recommendations: methodology and results summary. https://www.sleephealthjournal.org/article/S2352-7218(15)00015-7/fulltext.

^4^Working hours: ≤38 or >38 h/week. The Office for National Statistics. Average actual weekly hours of work for full-time workers (seasonally adjusted). https://www.ons.gov.uk/employmentandlabourmarket/peopleinwork/earningsandworkinghours/timeseries/ybuy/lms.

Descriptive statistics were used to summarize study population characteristics. Logistic regression analyses were undertaken to assess associations between IT worker status (compared to all other employed participants) and health or lifestyle outcomes. Model 0 was unadjusted for all covariates; Model 1 adjusted for potential confounders (age, sex, ethnicity, annual household income and socio-economic deprivation); Model 2 adjusted for both potential confounders (as above in Model 1) and mediators (smoking status, BMI, sleep duration and total screen-time, i.e. computer screen-time outside work plus TV-viewing time, where these were not the dependent variable). These regression models were repeated for the IT worker subgroups of managers, professionals, technicians (with managers as the reference category). Given potential differences between all other employed participants and the IT worker group, the models were also repeated for more comparable skill levels using the SOC category major group counterparts of functional managers (FMs), science and technology professionals (STPs) and science and technology associate professionals (STAPs), each as the reference categories, respectively.

Analyses were performed using statistical software Stata V14 (StataCorp LP). We analysed separately the study population for which most covariate values were available and then undertook a complete case analysis, which also included covariates with a higher proportion of missing data (i.e. alcohol consumption, physical activity and work/job satisfaction). This study was conducted under generic UK Biobank approval from NHS National Research Ethics service (Ref 11/NW/0382), Application number 17333.

## Results

**Figure 1.**
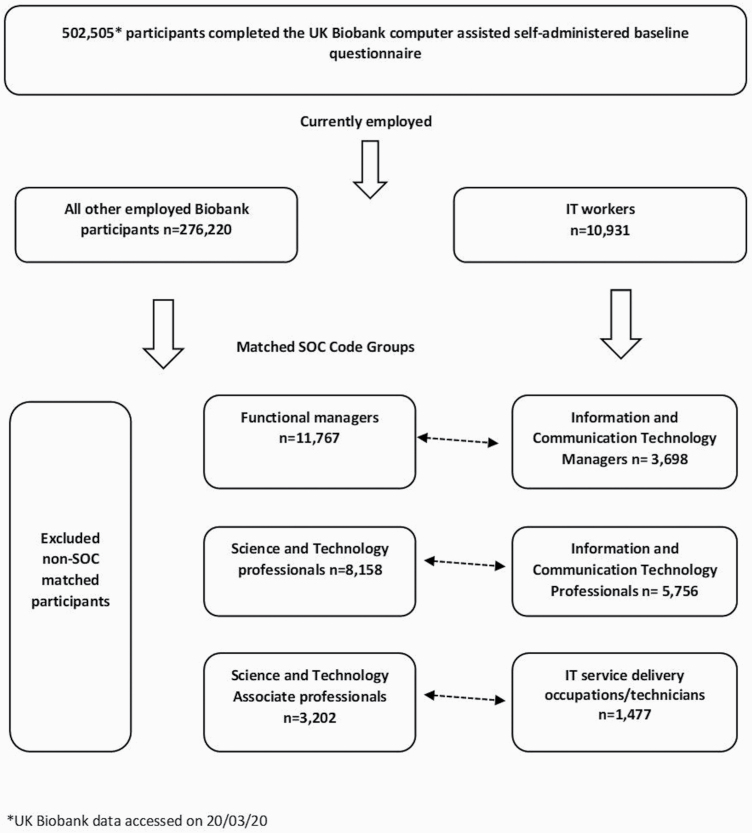
Flow chart of the selection process.

The stages in defining our study population are presented in Figure 1. Of the 287 151 employed participants included in this study, 10 931 (4%) were IT workers (Tables 1 and 2). Over three-quarters (76%) were male, with a median age of 50 years (25th/75th percentile: 45/55) (Table 1, column A). The IT workers, as compared to all other employed participants, were more likely to: hold a degree qualification (58% versus 37%), be least socio-economically deprived (52% versus 45%) and have an annual household income in excess of £52 000 (54% versus 31%). Conversely, they were less likely to be troubled with sleeplessness/insomnia compared to all other employed participants (34% versus 27% never/rarely affected, respectively).

**Table 1.** Socio-demographic and health characteristics (A) in IT workers compared to ‘all other employed’ participants in the UK Biobank and (B) within IT worker subgroups

	A		B		
	All other employed, *n* (%)	All IT workers, *n* (%)	IT managers, *n* (%)	IT professionals, *n* (%)	IT technicians, *n* (%)
Total *n* (%) 287 151 (100)	276 220 (96)	10 931 (4)	3698 (1)	5756 (2)	1477 (0.5)
Sex					
Male	129 601 (47)	8349 (76)	2809 (76)	4628 (80)	912 (62)
Female	146 619 (53)	2582 (24)	889 (24)	1128 (20)	565 (38)
Age (years), median (IQR; Q1/Q3)	53 (11; 47/58)	50 (10; 45/55)	50 (10; 45/55)	49 (11; 44/55)	51 (11; 45/56)
Age (years)					
40–44*	42 124 (15)	2642 (24)	871 (24)	1445 (25)	326 (22)
45–49	54 567 (20)	2707 (25)	945 (26)	1442 (25)	320 (22)
50–54	61 046 (22)	2570 (24)	896 (24)	1311 (23)	363 (25)
55–59	61 907 (22)	1876 (17)	665 (18)	934 (16)	277 (19)
60–64	44 593 (16)	983 (9)	284 (8)	536 (9)	163 (11)
65+	11 983 (4)	153 (1)	37 (1)	88 (2)	28 (2)
Ethnicity					
White	259 016 (94)	10 285 (94)	3525 (95)	5378 (93)	1382 (94)
Asian	5682 (2)	269 (3)	78 (2)	159 (3)	32 (2)
Black	5154 (2)	137 (1)	31 (1)	79 (1)	27 (2)
Chinese	1006 (0)	48 (0)	13 (0)	33 (1)	2 (0)
Mixed background/Others	4524 (2)	159 (2)	43 (1)	89 (2)	27 (2)
Missing**	838 (0)	33 (0)	8 (0)	18 (0)	7 (1)
Townsend deprivation index					
1 (least deprived quintile)	123 732 (45)	5695 (52)	2125 (58)	2892 (50)	678 (46)
2	62 874 (23)	2425 (22)	754 (20)	1331 (23)	340 (23)
3	42 470 (15)	1527 (14)	461 (13)	839 (15)	227 (15)
4	33 234 (12)	959 (9)	273 (7)	513 (9)	173 (12)
5 (most deprived quintile)	13 518 (5)	313 (3)	83 (2)	174 (3)	56 (4)
Missing**	392 (0)	12 (0)	2 (0)	7 (0)	3 (0)
Household annual income (£)					
Less than £18 000	27 053 (10)	207 (2)	27 (1)	112 (2)	68 (5)
£18 000 to £30 999	56 123 (20)	894 (8)	146 (4)	466 (8)	282 (19)
£31 000 to £51 999	77 824 (28)	3251 (30)	892 (24)	1796 (31)	563 (38)
£52 000 to £100 000	68 443 (25)	4717 (43)	1855 (50)	2471 (43)	391 (27)
Greater than £100 000	18 006 (7)	1128 (10)	579 (16)	509 (9)	40 (3)
Missing**	28 771 (10)	734 (7)	199 (5)	402 (7)	133 (9)
Highest qualification					
Degree,	101 264 (37)	6314 (58)	2111 (57)	3644 (63)	559 (38)
HNC/HND	19 067 (7)	590 (5)	199 (5)	271 (5)	120 (8)
School	111 416 (40)	3657 (34)	1273 (34)	1672 (29)	7132 (48)
Other	12 345 (5)	106 (1)	41 (1)	48 (1)	17 (1)
None of the above	28 072 (10)	144 (1)	43 (1)	50 (1)	51 (4)
Missing**	4056 (2)	120 (1)	31 (1)	71 (1)	18 (1)
Sleeplessness/insomnia					
Never/rarely	75 574 (27)	3698 (34)	1242 (34)	2008 (35)	448 (30)
Sometimes	132 105 (48)	5047 (46)	1704 (46)	2652 (46)	691 (47)
Usually	68 334 (25)	2179 (20)	750 (20)	1094 (19)	335 (23)
Missing**	207 (0)	7 (0)	2 (0)	2 (0)	3 (0)
Overall health (self-reported)					
Excellent/good	216 465 (78)	8794 (80)	3017 (82)	4646 (81)	1131 (77)
Fair/poor	58 862 (21)	2117 (19)	676 (18)	1098 (19)	343 (23)
Missing**	893 (0)	20 (0)	5 (0)	12 (0)	3 (0)

HNC, higher national certificate; HND, higher national diploma; IQR, interquartile range.

*35- to 39-year olds added to this total due to very small numbers *n* = 2.

**Includes ‘missing’, ‘do not know’ and ‘prefer not to answer’ responses.

IT workers comprised 3698 IT managers, 5756 IT professionals and 1477 IT technicians (1%, 2% and 0.5% of the total employed UK Biobank cohort, respectively)—see Table 1, column B. The median age of each subgroup was 50 years (25th/75th percentile: 45/55), 49 years (25th/75th percentile: 44/55) and 51 years (25th/75th percentile: 45/56), respectively, and in all groups the majority were male (76%, 80% and 62%, respectively). A higher proportion of managers were in the least socio-economically deprived category (58% versus 50% of professionals and 46% of technicians), yet a higher proportion of professionals held a degree qualification (63% versus 57% of managers and 38% of technicians).

**Figure 2.**
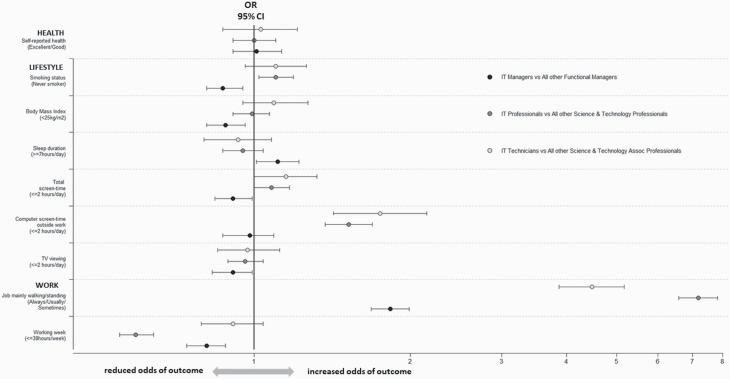
Multivariable logistic regression Model 2 (adjusted for potential confounders and mediators) of IT subgroups versus similar occupations within their SOC tree (functional Managers, science and technology Professionals, science and technology associate Professionals). Model 0: unadjusted and Model 1: adjusted for potential confounders, are included in Table S1 (available as Supplementary data at *Occupational Medicine* Online).

The logistic regression analyses in Table 3 were based on 252 932 individuals (88% of the total employed participants) and in Figure 2/Table S1 (available as Supplementary data at *Occupational Medicine* Online) were based on 14 363 FMs, 12 797 STPs and 4172 STAPs (93%, 92%, 89% of employed participants in these respective SOC trees).

**Table 3.** Multivariable logistic regression model results^∞^ for increased levels of risk factors (A) IT workers compared to all other employed Biobank participants and (B) IT worker subgroups

	A (reference category: *All other employed Biobank participants*)		B (reference category: *IT managers*)			
	All IT workers		IT professionals		IT technicians	
Total *n* (%)	287 151 (100)		10 931 (100)			
Total after missing data *n* (%)	252 932 (88)		10 094 (92)			
	OR	95% CI	OR	95% CI	OR	95% CI
Health						
Self-reported overall health (*excellent/good*^¥^)						
Model 0^a^	0.90	0.86–0.95	1.08	0.97–1.21	1.35	1.15–1.57
Model 1^b^	0.95	0.91–1.00	0.98	0.87–1.09	1.17	0.99–1.38
Model 2^c^	0.99	0.94–1.04	1.06	0.95–1.20	1.21	1.02–1.43
Lifestyle						
Smoking status (*never smoker*^¥^)						
Model 0^a^	0.80	0.76–0.83	0.87	0.80–0.95	1.06	0.93–1.21
Model 1^b^	0.85	0.81–0.88	0.84	0.77–0.92	0.94	0.82–1.08
Model 2^c^	0.85	0.82–0.89	0.86	0.78–0.94	0.94	0.82–1.08
BMI (*<25 kg/m*^*2*¥^)						
Model 0^a^	1.03	0.98–1.07	0.81	0.74–0.88	0.89	0.78–1.02
Model 1^b^	0.88	0.84–0.92	0.78	0.71–0.85	0.95	0.82–1.10
Model 2^c^	0.89	0.86–0.93	0.79	0.72–0.87	0.92	0.80–1.07
Sleep duration (*≥7 h/day*^¥^)						
Model 0^a^	0.96	0.92–1.00	0.86	0.78–0.95	1.00	0.87–1.16
Model 1^b^	0.99	0.95–1.04	0.83	0.75–0.91	0.94	0.81–1.10
Model 2^c^	1.00	0.96–1.05	0.84	0.76–0.93	0.93	0.80–1.09
Total screen-time, i.e. computer screen-time outside work + TV viewing *(≤2 h/day*^¥^)						
Model 0^a^	0.82	0.79–0.86	1.02	0.93–1.11	1.44	1.27–1.63
Model 1^b^	0.93	0.89–0.97	0.94	0.86–1.03	1.26	1.10–1.44
Model 2^c^	0.95	0.91–0.99	0.99	0.90–1.08	1.30	1.13–1.49
Computer screen-time outside work (*≤2 h/day*^¥^)						
Model 0^a^	1.66	1.57–1.76	1.19	1.05–1.33	1.04	0.87–1.25
Model 1^b^	1.40	1.33–1.49	1.07	0.95–1.21	0.93	0.77–1.13
Model 2^c^	1.42	1.35–1.51	1.11	0.98–1.26	0.95	0.78–1.14
TV viewing (*≤2 h/day*^¥^)						
Model 0^a^	0.69	0.66–0.72	0.95	0.87–1.04	1.50	1.32–1.71
Model 1^b^	0.81	0.78–0.85	0.89	0.81–0.98	1.34	1.17–1.53
Model 2^c^	0.83	0.79–0.87	0.93	0.85–1.03	1.38	1.20–1.58
Work						
Job involves mainly walking or standing (*always /usually/sometimes*^¥^)						
Model 0^a^	5.46	5.22–5.72	1.62	1.47–1.79	0.61	0.54–0.70
Model 1^b^	5.16	4.92–5.41	1.89	1.70–2.09	0.76	0.66–0.87
Model 2^c^	5.14	4.91–5.39	1.84	1.65–2.04	0.75	0.65–0.87
Working week (*≤38 h/week*^¥^)						
Model 0^a^	1.54	1.47–1.60	0.43	0.39–0.47	0.29	0.25–0.33
Model 1^b^	0.80	0.76–0.83	0.44	0.40–0.49	0.42	0.37–0.48
Model 2^c^	0.80	0.77–0.84	0.45	0.41–0.50	0.42	0.37–0.49

^∞^Variables with high ‘missing’ data, i.e. alcohol, physical activity and work/job satisfaction are not included here but in Table S1 (available as Supplementary data at *Occupational Medicine* Online).

*Italics* and ¥ denote the reference category. Model 0^a^ = unadjusted. Model 1^b^ = Model 0 + adjusted for confounders: age, sex, ethnicity, household annual income and deprivation. Model 2^c^ = Model 1 + potential mediators: smoking status, BMI, sleep duration and total screen-time (i.e. computer screen-time outside work plus TV-viewing time), where these are not the dependent variable.

IT workers were less likely to be previous/current smokers compared to all other employed participants even after adjustment (Model 2: odds ratio [OR] = 0.85, 95% confidence interval [CI]: 0.82–0.89) (Table 3, column A). IT professionals were less likely to be previous/current smokers compared to IT managers (Model 2: OR = 0.86, 95% CI: 0.78–0.94) (Table 3, column B).

After adjustment, IT workers had a reduced risk of a BMI in excess of recommended guidelines (Model 2: OR = 0.89, 95% CI: 0.86–0.93), compared to all other employed participants (Table 3, column A). IT professionals were less likely than IT managers to have an excessive BMI (Model 2: OR = 0.79, 95% CI: 0.72–0.87) (Table 3, column B).

For sleep duration, no substantial differences were seen between IT workers and all other employed participants, even after adjustment (Table 3, column A). IT professionals were less likely (Model 2: OR = 0.84, 95% CI: 0.76–0.93) to have inadequate sleep than IT managers (Table 3, column B).

IT workers were more likely to engage in >2 h/day of computer screen-time outside work compared to all other employed participants (Model 2: OR = 1.42, 95% CI: 1.35–1.51). IT professionals (Model 2: OR = 1.52, 95% CI: 1.37–1.69) and technicians (Model 2: OR = 1.75, 95% CI: 1.42–2.15) were more likely to engage in >2 h/day of computer screen-time outside work than their respective STP and STAP counterparts, although no substantive differences were seen for IT managers (Figure 2).

The odds of IT worker’s jobs being sedentary were over five times compared to all other employed participants, even after adjustment (Model 2: OR = 5.14, 95% CI: 4.91–5.39) (Table 3, column A). Across all models and attenuated after adjustment, the odds of IT professional’s jobs being sedentary were almost two times that of IT managers (Model 2: OR = 1.84, 95% CI: 1.65–2.04). Conversely, IT technician’s jobs were less sedentary compared to IT managers (Model 2: OR = 0.75, 95% CI: 0.65–0.87) (Table 3, column B). Across all models and with attenuation after adjustment for managers and professionals, the odds of IT manager’s jobs being sedentary were approximately two times (Model 2: OR = 1.83, 95% CI: 1.68–1.99); IT professional’s jobs were over seven times (Model 2: OR = 7.18, 95% CI: 6.58–7.82); and IT technician’s jobs were over four times (Model 2: OR = 4.48, 95% CI: 3.87–5.17), that of their respective FM, STP and STAP counterparts (Figure 2).

IT workers were less likely to work beyond the average working week (Model 2: OR = 0.80, 95% CI: 0.77–0.84) compared to all other employed participants (Table 3, column A). IT professionals (Model 2: OR = 0.45, 95% CI: 0.41–0.50) and technicians (Model 2: OR = 0.42, 95% CI: 0.37–0.49) were substantially less likely to work beyond the average working week compared to IT managers (Table 3, column B). IT managers (Model 2: OR = 0.81, 95% CI: 0.74–0.88) and IT professionals (Model 2: OR = 0.59, 95% CI: 0.55–0.64) were both less likely to work beyond the average working week compared to their respective counterparts (Figure 2).

All other associations are shown in Table 3, Figure 2 and Table S1 (available as Supplementary data at *Occupational Medicine* Online). Although alcohol, physical activity and work/job satisfaction were variables limited by a high proportion of missing data, and the results therefore to be considered with caution, we observed the following findings for these outcomes (analyses were based on 34 278 individuals, i.e. 12% of the total employed participants).

For alcohol consumption, after adjustment, no differences were found for IT workers compared to all other employed participants or across IT worker subgroups (Table S2, columns A and B, available as Supplementary data at *Occupational Medicine* Online).

Across all three models an increased risk (Model 2: OR = 1.26, 95% CI: 1.11–1.43) of inadequate physical activity in IT workers compared to all other employed Biobank participants was observed. No differences were found across IT worker subgroups (Table S2, columns A and B, available as Supplementary data at *Occupational Medicine* Online).

Across all three models an increased likelihood of IT workers being unhappy in their work/jobs (Model 2: OR = 1.67, 95% CI: 1.45–1.93) compared to all other employed Biobank participants was observed. No differences were found across IT worker subgroups (Table S2, columns A and B, available as Supplementary data at *Occupational Medicine* Online).

## Discussion

In this, the largest to date study of IT worker health, IT workers have substantially higher exposures to sedentary work than both all other employed participants (five times the odds) and their SOC group counterparts (approximately two, seven and four times the odds, for managers, professionals and technicians, respectively). IT workers reported similar overall health, but lower lifestyle risk factors for smoking and obesity. IT professionals are less likely to have lifestyle risks of smoking, obesity and inadequate sleep than IT managers yet conversely, their jobs are substantially more sedentary. IT technicians’ jobs are less sedentary compared to IT managers. This is likely related to their technical services support role, which can be peripatetic, responding to helpdesk calls, particularly for those not working in IT companies.

This UK-based study is substantially larger than any previously published studies examining IT worker health and well-being. It has a large range and rich characterization of variables. In contrast to other studies, it is not restricted to a specific IT company. With recruitment from the general working population, it includes IT workers working in different IT companies and those employed out with IT companies (across various organizations/sectors), thereby providing a broader and more generalizable overview of IT worker health and well-being. Additionally, it fills a knowledge gap of risks related to NCDs in IT workers.

The UK Biobank was set up for a broader purpose than specifically evaluating occupational factors, so there is limited OH and exposure information and no sickness absence data. Response rates to the baseline UK Biobank survey are acknowledged to be low (https://www.ukbiobank.ac.uk/), participants are aged ≥40 years and our study included those employed only. Therefore, our results may not be representative of all UK IT workers. Potential selection bias through the recruitment method, recall bias and healthy-worker effect may underestimate the true occupational risks of IT work and the cross-sectional nature of this study is more about describing health needs, rather than establishing causality.

While our results are consistent with the broader literature for some risk factors, there is variance for others. Our findings of less previous/current smoking and higher income levels are in keeping with those of an Indian software industry study [17]. A 1983 US IT professional study [9] which assessed alcohol and smoking precludes comparison with our findings, due to variable heterogeneity and the historical nature of the data. Sleep deficit and sleeplessness/insomnia not identified as substantive factors in our IT population are consistent with a Finnish study [5], where sleep debt and insomnia were not common in their IT population. A study [11] of Indian and US-based software professionals observed that getting ‘good’ sleep was the commonest strategy used to manage health and work-related symptoms. Conversely, in another Indian study [18], over half reported insomnia symptoms. A Taiwanese study [16] reported a higher obesity prevalence among IT workers, whereas our study identified a lower comparative obesity risk. A general population review identified limited evidence of a longitudinal relationship between sedentary behaviour and obesity risk in adults [22]. Sedentary behaviour, however, has been identified as a distinct risk factor (independent of physical activity), for multiple adverse health outcomes including: increased all-cause mortality, cardiovascular disease, cancer, diabetes, metabolic syndrome and mental illness [23,24]. Our working week findings are in contrast with Finnish and Japanese studies [5,25], which report excessive working hours in IT employees. Our results could demonstrate differing work cultures and conditions across these countries. This is supported by a study comparing Indian and US employees of the same IT company, with the Indian employees reporting a 10.5-h and the US employees reporting a 7.5-h average working day [11].

While IT has brought us physical activity tracking devices and prompting apps (reminding us to get up and move regularly), our findings highlight that IT workers themselves are not adopting this important health promotion advice. There is a need for interventions focused on reducing sedentary behaviour both within and out with the workplace and for education and awareness among IT workers to minimize sedentary behaviour.

Despite some evidence from our study that IT workers, IT professionals in particular, either intentionally or unintentionally adopt other positive health and lifestyle measures, the overall impact of sedentary behaviour on IT worker health is unclear. Further research on disease incidence (particularly those associated with sedentary behaviour such as cardio-metabolic disease and cancer) in IT workers is needed to evaluate the health impact of this risk factor. The higher computer screen-time outside work among IT workers identified in our study may represent leisure use, but it may also potentially reflect an extension of work beyond the standard working day/hours and merits further research.

This study sets a benchmark for large-scale IT worker health studies. Improved understanding of health, lifestyle and occupational risk factors of this large and mixed IT worker population can help inform workplace interventions to mitigate risk, improve health and increase the work participation of this rapidly growing and under-researched occupational group.

## Supplementary Material

kqaa222_suppl_Supplementary-Table-S1_and_S2
