# Supplementary material for: Health, lifestyle and occupational risks in Information Technology workers
Source: Occup Med (Lond). 2021 Jan 30;71(2):68–74. doi: 10.1093/occmed/kqaa222 (PMC8034523; doi:10.1093/occmed/kqaa222)
Supplement: kqaa222_suppl_Supplementary-Table-S1_and_S2 [file kqaa222_suppl_supplementary-table-s1_and_s2.docx]

**Supplementary Table S1. Multivariable logistic regression model results^∞^ for increased levels of risk factors. Comparison of IT sub-groups with other similar occupations within their SOC tree. A) Functional Managers B) Science and Technology Professionals C) Science and Technology Associate Professionals.**

|  | **A** (Reference Category: *All other Functional managers^¥^)* | | **B** (Reference Category: *All other Science &Technology professionals^¥^)* | | **C** (Reference Category: *All other Science & Technology Associate professionals^¥^)* | |
| --- | --- | --- | --- | --- | --- | --- |
|  | **IT managers** | | **IT professionals** | | **IT technicians** | |
| Total n (%)  Total after missing data n (%) | 15,465 (100)  14,363 (93) | | 13,914 (100)  12,797 (92) | | 4679 (100)  4,172 (89) | |
|  | OR | 95% CI | OR | 95% CI | OR | 95% CI |
| **Health**  Self-reported overall health *(Excellent/Good ^¥^)*  Model 0 ^a^  Model 1 ^b^  Model 2 ^c^  **Lifestyle**  Smoking status *(Never smoker ^¥^)*  Model 0 ^a^  Model 1 ^b^  Model 2 ^c^  Body Mass Index *(<25kg/m2 ^¥^)*  Model 0 ^a^  Model 1 ^b^  Model 2 ^c^  Sleep duration *(≥7hours /day ^¥^)*  Model 0 ^a^  Model 1 ^b^  Model 2 ^c^  Total screen-time i.e. computer screen-time outside work + TV viewing *(≤2 hours/day ^¥^)*  Model 0 ^a^  Model 1 ^b^  Model 2 ^c^  Computer screen-time outside work *(≤2 hours/day ^¥)^*  Model 0 ^a^  Model 1 ^b^  Model 2 ^c^  TV viewing *(≤2 hours /day ^¥)^*  Model 0 ^a^  Model 1 ^b^  Model 2 ^c^  **Work**  Job involves mainly walking or standing  *(Always /Usually/Sometimes^¥^)*  Model 0 ^a^  Model 1 ^b^  Model 2 ^c^  Working week *(≤38hours/week ^¥^)*  Model 0 ^a^  Model 1 ^b^  Model 2 ^c^ | 1.04  0.85  1.01  0.84  0.87  0.87  1.00  0.87  0.88  1.11  1.09  1.11  0.91  0.90  0.91  1.01  0.97  0.98  0.89  0.89  0.91  1.74  1.84  1.83  1.05  0.81  0.81 | 0.94-1.15  0.64-1.12  0.91-1.13  0.77-0.91  0.80-0.94  0.81-0.95  0.92-1.08  0.80-0.95  0.81-0.96  1.01-1.21  1.00-1.20  1.01-1.22  0.84-0.98  0.83-0.97  0.84-0.99  0.91-1.13  0.87-1.09  0.87-1.09  0.82-0.96  0.82-0.97  0.83-0.99  1.60-1.89  1.69-2.00  1.68-1.99  0.96-1.14  0.74-0.89  0.74-0.88 | 1.02  1.02  1.00  1.01  1.10  1.10  0.93  1.01  0.99  0.96  0.96  0.95  1.00  1.09  1.08  1.52  1.52  1.52  0.88  0.97  0.96  6.81  7.00  7.18  0.70  0.59  0.59 | 0.94-1.12  0.93-1.12  0.91-1.10  0.94-1.09  1.03-1.20  1.02-1.19  0.86-1.00  0.93-1.09  0.91-1.07  0.89-1.05  0.88-1.05  0.87-1.04  0.94-1.08  1.01-1.17  1.00-1.17  1.37-1.68  1.37-1.69  1.37-1.69  0.81-0.94  0.90-1.04  0.89-1.04  6.27-7.39  6.42-7.62  6.58-7.82  0.65-0.75  0.55-0.64  0.55-0.64 | 1.02  1.04  1.03  1.02  1.11  1.10  1.03  1.12  1.09  0.93  0.94  0.93  1.09  1.17  1.15  1.79  1.75  1.75  0.92  0.99  0.97  4.47  4.41  4.48  0.95  0.90  0.91 | 0.87-1.19  0.89-1.22  0.87-1.21  0.89-1.16  0.96-1.27  0.96-1.26  0.90-1.19  0.97-1.29  0.95-1.27  0.80-1.08  0.81-1.09  0.80-1.08  0.96-1.24  1.01-1.33  1.00-1.32  1.47-2.19  1.42-2.15  1.42-2.15  0.81-1.05  0.86-1.13  0.85-1.12  3.89-5.13  3.82-5.09  3.87-5.17  0.83-1.08  0.79-1.04  0.79-1.04 |

∞ Variables with high ‘missing’ data i.e. alcohol, physical activity and work/job satisfaction are not included here but in Supplementary Table 4.

*Italics* and ¥ denote the reference category

OR, odds ratio; CI, confidence interval.

Model 0 ^a^ = Unadjusted

Model 1 ^b^ = Model 0 + adjusted for confounders: age, sex, ethnicity, household annual income and deprivation.

Model 2 ^c^ = Model 1 + potential mediators: smoking status, body mass index, sleep duration and total screen-time (i.e. computer screen-time outside work plus TV viewing time), where these are not the dependent variable.

**Supplementary Table S2. Multivariable logistic regression model results for increased levels of risk factors, including variables with high ‘missing’ data i.e. alcohol, physical activity and work/job satisfaction. A) IT workers compared to all other employed Biobank participants and B) IT worker sub-groups.**

|  | A (Reference Category-All other employed Biobank participants ^¥^) | | B (Reference Category: IT managers^¥^) | | | |
| --- | --- | --- | --- | --- | --- | --- |
|  | **All IT workers** | | **IT professionals** | | **IT technicians** | |
| Total n (%)  Total after missing data n (%) | 287,151 (100)  34,278 (12) | | 10,931 (100)  1,458 (13) | | | |
|  | OR | 95% CI | OR | 95% CI | OR | 95% CI |
| **Health**  Self-reported overall health *(Excellent/Good ^¥^)*  Model 0 ^a^  Model 1 ^b^  Model 2 ^c^  **Lifestyle**  Smoking status *(Never smoker ^¥^)*  Model 0 ^a^  Model 1 ^b^  Model 2 ^c^  Alcohol consumption*** (*≤14 units/week* ^¥)^  Model 0 ^a^  Model 1 ^b^  Model 2 ^c^  Body Mass Index *(<25kg/m2 ^¥^)*  Model 0 ^a^  Model 1 ^b^  Model 2 ^c^  Sleep duration *(≥7hours /day ^¥^)*  Model 0 ^a^  Model 1 ^b^  Model 2 ^c^  Physical activity (*≥600 MET min/week*^¥^)  Model 0 ^a^  Model 1 ^b^  Model 2 ^c^  Total screen-time i.e. computer screen-time outside work + TV viewing *(≤2 hours/day ^¥^)*  Model 0 ^a^  Model 1 ^b^  Model 2 ^c^  Computer screen-time outside work *(≤2 hours/day ^¥)^*  Model 0 ^a^  Model 1 ^b^  Model 2 ^c^  TV viewing *(≤2 hours /day ^¥)^*  Model 0 ^a^  Model 1 ^b^  Model 2 ^c^  **Work**  Work/job satisfaction (*Happy*^¥^)  Model 0 ^a^  Model 1 ^b^  Model 2 ^c^  Job involves mainly walking or standing  *(Always /Usually/Sometimes^¥^)*  Model 0 ^a^  Model 1 ^b^  Model 2 ^c^  Working week *(≤38hours/week ^¥^)*  Model 0 ^a^  Model 1 ^b^  Model 2 ^c^ | 0.94  0.90  0.82  0.82  0.88  0.89  1.15  0.91  0.93  1.05  0.84  0.85  1.12  1.12  1.09  1.35  1.28  1.26  0.87  0.91  0.92  1.54  1.32  1.34  0.74  0.81  0.81  1.89  1.68  1.67  4.83  4.81  4.66  1.45  0.78  0.78 | 0.81-1.08  0.77-1.04  0.70-0.95  0.74-0.91  0.79-0.98  0.80-1.00  1.02-1.29  0.80-1.03  0.82-1.05  0.94-1.17  0.75-0.94  0.76-0.95  1.00-1.26  1.00-1.27  0.97-1.23  1.20-1.53  1.13-1.46  1.11-1.43  0.78-0.97  0.82-1.02  0.82-1.02  1.33-1.78  1.14-1.53  1.16-1.56  0.66-0.83  0.72-0.91  0.72-0.92  1.65-2.17  1.46-1.94  1.45-1.93  4.29-5.42  4.26-5.43  4.12-5.27  1.30-1.61  0.69-0.87  0.69-0.87 | 1.14  1.04  1.16  0.84  0.80  0.82  0.93  0.98  1.03  0.65  0.61  0.62  0.84  0.78  0.82  0.95  0.92  0.95  1.20  1.11  1.17  1.35  1.27  1.29  1.18  1.10  1.18  1.13  1.11  1.13  1.72  2.16  2.04  0.47  0.49  0.50 | 0.84-1.56  0.76-1.43  0.82-1.63  0.67-1.05  0.63-1.01  0.65-1.04  0.72-1.20  0.75-1.27  0.78-1.34  0.52-0.82  0.48-0.77  0.48-0.79  0.66-1.09  0.60-1.02  0.69-0.98  0.73-1.23  0.70-1.19  0.73-1.24  0.96-1.52  0.88-1.41  0.92-1.49  0.99-1.85  0.92-1.75  0.93-1.78  0.92-1.50  0.86-1.42  0.91-1.52  0.84-1.51  0.82-1.50  0.83-1.53  1.33-2.22  1.65-2.84  1.55-2.68  0.37-0.59  0.38-0.62  0.39-0.64 | 1.41  1.27  1.21  1.18  1.00  0.98  0.83  0.96  0.98  0.75  0.79  0.76  1.17  0.96  0.85  0.71  0.74  0.76  1.64  1.46  1.47  1.38  1.23  1.25  1.52  1.43  1.45  1.17  1.26  1.23  0.49  0.64  0.59  0.40  0.55  0.55 | 0.89-2.25  0.78-2.08  0.71-2.05  0.82-1.68  0.69-1.46  0.67-1.45  0.56-1.24  0.63-1.46  0.64-1.49  0.52-1.09  0.53-1.16  0.51-1.13  0.80-1.72  0.64-1.45  0.65-1.11  0.45-1.10  047-1.17  0.48-1.20  1.15-2.34  1.00-2.13  1.00-2.14  0.86-2.23  0.75-2.03  0.76-2.08  1.05-2.21  0.97-2.12  0.97-2.16  0.74-1.85  0.78-2.04  0.76-2.01  0.34-0.70  0.43-0.94  0.40-0.88  0.28-0.57  0.37-0.82  0.37-0.82 |

*Italics* and ¥ denote the reference category

OR, odds ratio; CI, confidence interval.

Model 0 ^a^ = Unadjusted

Model 1 ^b^ = Model 0 + adjusted for confounders: age, sex, ethnicity, household annual income and deprivation.

Model 2 ^c^ = Model 1 + potential mediators: smoking status, body mass index, sleep duration and total screen-time (i.e. computer screen-time outside work plus TV viewing time), where these are not the dependent variable.

***The recommended alcohol consumption guidelines changed in 2016 (i.e. following baseline data collection) from 21 units/week for women and 28 units/week for men to these current thresholds of 14 units/week for men and women.
